# RETRACTION: Combination of Aloin and Metformin Enhances the Antitumor Effect by Inhibiting the Growth and Invasion and Inducing Apoptosis and Autophagy in Hepatocellular Carcinoma Through PI3K/AKT/mTOR Pathway

**DOI:** 10.1002/cam4.70055

**Published:** 2024-08-14

## Abstract

**RETRACTION:** R. Sun, R. Zhai, C. Ma, and W. Miao, “Combination of Aloin and Metformin Enhances the Antitumor Effect by Inhibiting the Growth and Invasion and Inducing Apoptosis and Autophagy in Hepatocellular Carcinoma Through PI3K/AKT/mTOR Pathway,” *Cancer Medicine* 9, no. 3 (2020): 1141–1151, https://doi.org/10.1002/cam4.2723.

The above article, published online on 12 December 2019 in Wiley Online Library (wileyonlinelibrary.com), has been retracted by agreement between the authors; the journal Editor‐in‐Chief, Stephen Tait; and John Wiley and Sons Ltd. The authors asked to retract the article as they found significant errors in the data collection and analysis, therefore the conclusions of the paper are not supported. Subsequently, an investigation into concerns raised by a third party revealed inappropriate duplication of images (Figure 1C, 2B and 4C, D) between this and other articles that were previously published or published shortly after in a different scientific context. Thus, the editors consider the conclusions of this manuscript substantially compromised. Although the authors initially asked for the retraction, when asked to approve the retraction wording, they remained unresponsive.

**RETRACTION:** R. Sun, R. Zhai, C. Ma, and W. Miao, “Combination of Aloin and Metformin Enhances the Antitumor Effect by Inhibiting the Growth and Invasion and Inducing Apoptosis and Autophagy in Hepatocellular Carcinoma Through PI3K/AKT/mTOR Pathway,” *Cancer Medicine* 9, no. 3 (2020): 1141–1151, https://doi.org/10.1002/cam4.2723.

The above article, published online on 12 December 2019 in Wiley Online Library (wileyonlinelibrary.com), has been retracted by agreement between the authors; the journal Editor‐in‐Chief, Stephen Tait; and John Wiley and Sons Ltd. The authors asked to retract the article as they found significant errors in the data collection and analysis, therefore the conclusions of the paper are not supported. Subsequently, an investigation into concerns raised by a third party revealed inappropriate duplication of images (Figure 1C, 2B and 4C, D) between this and other articles that were previously published or published shortly after in a different scientific context. Thus, the editors consider the conclusions of this manuscript substantially compromised. Although the authors initially asked for the retraction, when asked to approve the retraction wording, they remained unresponsive.

